# Gun Violence Exposure and Suicide Among Black Adults

**DOI:** 10.1001/jamanetworkopen.2023.54953

**Published:** 2024-02-06

**Authors:** Daniel C. Semenza, Samantha Daruwala, Jasmin R. Brooks Stephens, Michael D. Anestis

**Affiliations:** 1 Department of Sociology, Anthropology, and Criminal Justice, Rutgers University, Camden, New Jersey; 2 Department of Urban-Global Public Health, Rutgers University, Piscataway, New Jersey; 3 New Jersey Gun Violence Research Center, Rutgers University, Piscataway; 4 VA Center of Excellence for Suicide Prevention, VA Finger Lakes Health Care System, Canandaigua, New York; 5 Department of Psychiatry and Behavioral Health, The Ohio State University Wexner Medical Center, Columbus; 6 Department of Psychiatry, Massachusetts General Hospital, Boston

## Abstract

**Importance:**

Black individuals are disproportionately exposed to gun violence in the US. Suicide rates among Black US individuals have increased in recent years.

**Objective:**

To evaluate whether gun violence exposures (GVEs) are associated with suicidal ideation and behaviors among Black adults.

**Design, Setting, and Participants:**

This cross-sectional study used survey data collected from a nationally representative sample of self-identified Black or African American (hereafter, Black) adults in the US from April 12, 2023, through May 4, 2023.

**Exposures:**

Ever being shot, being threatened with a gun, knowing someone who has been shot, and witnessing or hearing about a shooting.

**Main Outcomes and Measures:**

Outcome variables were derived from the Self-Injurious Thoughts and Behaviors Interview, including suicidal ideation, suicide attempt preparation, and suicide attempt. A subsample of those exhibiting suicidal ideation was used to assess for suicidal behaviors.

**Results:**

The study sample included 3015 Black adults (1646 [55%] female; mean [SD] age, 46.34 [0.44] years [range, 18-94 years]). Most respondents were exposed to at least 1 type of gun violence (1693 [56%]), and 300 (12%) were exposed to at least 3 types of gun violence. Being threatened with a gun (odds ratio [OR], 1.44; 95% CI, 1.01-2.05) or knowing someone who has been shot (OR, 1.44; 95% CI, 1.05-1.97) was associated with reporting lifetime suicidal ideation. Being shot was associated with reporting ever planning a suicide (OR, 3.73; 95% CI, 1.10-12.64). Being threatened (OR, 2.41; 95% CI, 2.41-5.09) or knowing someone who has been shot (OR, 2.86; 95% CI, 1.42-5.74) was associated with reporting lifetime suicide attempts. Cumulative GVE was associated with reporting lifetime suicidal ideation (1 type: OR, 1.69 [95% CI, 1.19-2.39]; 2 types: OR, 1.69 [95% CI, 1.17-2.44]; ≥3 types: OR, 2.27 [95% CI, 1.48-3.48]), suicide attempt preparation (≥3 types; OR, 2.37; 95% CI, 2.37-5.63), and attempting suicide (2 types: OR, 4.78 [95% CI, 1.80-12.71]; ≥3 types: OR, 4.01 [95% CI, 1.41-11.44]).

**Conclusions and Relevance:**

In this cross-sectional study, GVE among Black adults in the US was significantly associated with lifetime suicidal ideation and behavior. Public health efforts to substantially reduce interpersonal gun violence may yield additional benefits by decreasing suicide among Black individuals in the US.

## Key Points

**Question:**

Are individual and cumulative forms of gun violence exposure (GVE) associated with suicidal ideation and behaviors among Black adults in the US?

**Findings:**

In this cross-sectional study of 3015 US Black adults, both individual and cumulative GVEs were significantly associated with lifetime suicidal ideation, suicide attempt preparation, and attempting suicide.

**Meaning:**

Reducing GVE may be necessary to address rising rates of suicide among Black individuals in the US.

## Teaser

This cross-sectional study examines whether individual and cumulative forms of gun violence exposure are associated with suicidal ideation and behaviors in Black adults in the US.

## Introduction

In 2021, almost 49 000 individuals died due to gun violence in the US, representing the highest number of annual gun-related deaths ever recorded.^1^ Additionally, nearly 85 000 additional nonfatal shootings occur each year.^2,3^ The rate of firearm injuries that require inpatient admission among Black individuals is 9 times higher than for their White counterparts.^4^ Black individuals in the US are almost 14 times more likely than their White counterparts to die due to firearm homicide.^1^ High rates of interpersonal gun violence are the product of past and present forms of discrimination, including racial segregation, concentrated poverty, mass incarceration, and lack of social mobility, which disproportionately affect Black individuals in the US.^5,6,7,8,9^

Suicide rates have increased dramatically among Black individuals in the US in recent years. From 2018 to 2021, the age-adjusted overall suicide rate increased by 37% among Black children, adolescents, and young adults (aged 10-24 years), and there was a 23% increase among younger Black adults (aged 25-44 years).^10^ Among Black individuals in the US, the age-adjusted firearm suicide rate increased by 44% from 2019 to 2021.^1^ Provisional data from 2022 show the firearm suicide rate among Black individuals exceeded the rate of their White peers for the first time in history, underscoring an urgent need to investigate this issue among both children and adults.^11^

Few studies have examined the association between interpersonal gun violence exposure (GVE) and risk for suicide, although evidence indicates that GVE is associated with worse mental health outcomes for those who experience it.^12^ Individuals who survive a gunshot are more likely to experience posttraumatic stress disorder, hypervigilance, and increased substance use.^13,14,15^ Those secondarily exposed to gun violence through the death of someone they know experience significantly higher levels of psychological distress and depression, with an increased likelihood of suicidal ideation and psychosis-like experiences.^16^ Furthermore, the prevalence of mental health diagnoses increases among both youths and adults in the 12 months after a family member’s homicide or a nonfatal shooting.^17,18^ It is especially important to consider the role of GVE in shaping mental health outcomes among Black individuals because they are more likely to be ascribed symptoms of mental illness and diagnoses compared with their White peers, affecting clinical prognoses and exacerbating racial disparities in health care.^19,20^

Gun violence contributes to individual, family, and collective trauma, and Black individuals in the US are more likely than other racial groups to live in neighborhoods with high rates of gun violence.^21,22,23^ Exposure to local homicide acutely interrupts children’s cognitive performances, attention, and impulse control.^24,25^ Greater proximity to shootings is also associated with increased risk for depression, especially among Black boys living in disadvantaged communities.^26^ Systematic racial discrimination not only affects the risk for exposure to gun violence and health outcomes among Black individuals in the US but also inhibits access to high-quality health care that ameliorates harms associated with GVE.

Although GVE is associated with mental health outcomes,^13,14,15,16,17,18^ there remain limitations to extant research. First, research tends to focus on mental health–related symptoms of anxiety, depression, and posttraumatic stress disorder, and there are few studies regarding how GVE is associated with suicidal ideation and behavior.^16,27^ It also remains unclear how particular types of GVE (eg, direct, secondary, and community) are associated with suicidal outcomes. Second, little is understood about the association between cumulative exposure to gun violence and suicide. Finally, research is needed to specifically investigate this issue among Black individuals while accounting for differences in the structural, personal, and socioeconomic factors that proxy systemic discrimination and are associated with both GVE and suicide-related outcomes.^1,11^ This work is necessary to inform public health strategies, including primary violence prevention and victim service provision, to reduce rising suicide rates among Black individuals in the US.

## Methods

### Data

In this cross-sectional study, we conducted a nationally representative survey of self-identified Black or African American (hereafter, Black) adults in the US in April and May 2023. The survey was disseminated by Ipsos Public Affairs using KnowledgePanel, the largest probability-based web panel representative of US adults.^28^ Following completion of a brief initial survey to determine racial identification, Ipsos KnowledgePanel members were invited via email to complete the survey. Informed consent was obtained from all study participants through an online form, and the survey was approved by the institutional review board at Rutgers University. This study followed the Strengthening the Reporting of Observational Studies in Epidemiology (STROBE) reporting guideline for cross-sectional studies.

We used poststratification weights to ensure that the sample was representative of Black adults in the US. Weights were created by weighting the respondent pool to geodemographic benchmarks that combine a supplement of the Current Population Survey with the US Census Bureau’s American Community Survey. Qualified respondents were weighted to geodemographic distributions for household income, sex, race, census region, metropolitan status, and educational level. An iterative raking procedure across all distributions was used to produce the final weights, which were scaled and trimmed to generate the final sample.

### Measures

#### Outcomes

We measured suicidal ideation using 8 items from the self-report version of the Self-Injurious Thoughts and Behaviors Interview.^29^ Respondents were asked, “Have you ever had any of the following thoughts for more than a few minutes? Choose all that apply.” The items included the following: “I wish I could disappear or not exist,” “I wish I was never born,” “My life is not worth living,” “I wish I could go to sleep and never wake up,” “I wish I were dead,” “Maybe I should kill myself,” “I should kill myself,” and “I’m going to kill myself.” We summed all items (α = 0.88) and created a binary measure of whether the individual had ever had any suicidal thoughts. Respondents were also asked whether they had experienced these thoughts within the past year or month. We created binary indicators of lifetime, past-year, and past-month suicidal ideation.

We measured suicide attempt preparation if respondents indicated they had done something to prepare a suicide attempt, such as gaining access to a method or writing a suicide note. These specific behaviors differ from having a fully realized, coherent suicide plan. We measured attempted suicide if respondents indicated they had tried to kill themselves.

#### Exposures

We used 4 items to measure GVE. We used the words *gun* and *firearm* interchangeably, acknowledging slight definitional differences between the 2. Shot with a firearm was measured using the question, “Have you ever been shot on purpose by another person with a firearm?” Threatened with a firearm was measured using the question, “Have you ever been threatened with a firearm by another person?” Family or friend shot was measured with the question, “Do you personally know someone such as a friend or family member who has been shot on purpose by another person with a firearm?” We measured witnessed or heard about a shooting using the question, “Have you ever witnessed or heard about someone being shot intentionally by another person with a firearm in your neighborhood?” We did not specify a definition for neighborhood to allow respondents to answer based on their own definition. Respondents answered “yes” or “no” for all 4 items.

We summed the 4 GVE measures to create a scale of cumulative GVE. We combined respondents exposed to 3 and 4 types of firearm violence into a single group of those who had experienced 3 or more types of exposure.

#### Covariates

All models accounted for covariates, including sex (male, female), self-rated health (poor, fair, good, very good, or excellent), age (18-29, 30-44, 45-59, or ≥60 years), educational level (no high school, high school degree, some college, or bachelor’s degree or more), household income (<$24 999, $25 000-$74 999, $75 000-$149 999, or≥$150 000), marital status (married, widowed, divorced, separated, or never married), employment status (working full-time, working part-time, or not working), current or former military status (yes, no), health insurance (yes, no), metropolitan area residence (yes, no), number of children living in the home, and US region of residence (Northeast, Midwest, South, or West).

### Statistical Analysis

We analyzed patterns of missingness, and the Little Test for Missing Completely at Random indicated that a small amount of data was missing completely at random (probability > χ^2^ = 0.4944). We used listwise deletion to account for missing cases in the main sample after including all covariates (91 [3%]). We compared results with multiply imputed data using chained equations (50 imputation sets), and results were substantively identical. All imputed results are available on request.

We used logistic regression models to examine the association between individual types of GVE and suicidal ideation (lifetime, past year, or past month). We analyzed associations between individual GVEs and suicidal behaviors (attempt preparation or attempted suicide) in a subsample of respondents who indicated having ever experienced suicidal thoughts. The subsample analysis aligned with ideation-to-action theories of suicide to ensure that we captured the association between exposures and specific suicidal behaviors, rather than simply proxying actual behaviors for suicidal ideation in the full sample.^30,31^ We repeated these steps to analyze associations between cumulative GVE and all outcomes. We interpreted statistical significance at the 2-tailed *P* < .05 level. Analysis was performed using Stata 18.0 (StataCorp LLC).

## Results

In total, 7133 total respondents were fielded, and 4234 completed the survey (59% completion rate). Ultimately, 3015 respondents who identified as Black were included in the final sample. Mean (SD) age was 46.34 (0.44) years (range, 18-94 years); 1646 (55%) were female, 1369 (45%) were male, 972 (32%) were aged 30 to 44 years, 1710 (57%) reported at least some college education, and 1700 (57%) lived in the South. Table 1 gives all weighted descriptive statistics. About a quarter of the sample (689 [23%]) reported lifetime suicidal ideation, 377 (13%) reported suicidal ideation in the past year, and 167 (6%) reported suicidal ideation in the past month. A total of 160 (5%) indicated ever making preparations for a suicide attempt, while 138 (4%) indicated attempting suicide. Most respondents (1693 [56%]) were exposed to at least 1 form of gun violence. The most common type of GVE was knowing someone who had been shot (1237 [41%]) followed by witnessing or hearing about a shooting (1138 [38%]). Notably, 300 respondents (12%) reported being exposed to 3 or more types of gun violence.

**Table 1.** Weighted Descriptive Statistics for the Full Sample

Variable	Respondents, No. (%) (N = 3015)
Suicidal ideation	
Lifetime	689 (23)
Past year	377 (13)
Past month	167 (6)
Suicide attempt preparation	160 (5)
Attempted suicide	138 (4)
Gun violence exposure types	
Threatened with a gun	649 (22)
Shot with a gun	80 (3)
Family or friend shot	1237 (41)
Witnessed or heard about a shooting	1138 (38)
Cumulative gun violence exposure	
0	1230 (41)
1	789 (27)
2	604 (20)
≥3	300 (12)
Sex	
Female	1646 (55)
Male	1369 (45)
Self-rated health	
Poor	75 (2)
Fair	607 (21)
Good	1262 (42)
Very good	789 (27)
Excellent	264 (9)
Age, y	
18-29	562 (19)
30-44	972 (32)
45-59	728 (24)
≥60	754 (25)
Educational level	
No high school	177 (6)
High school degree	1128 (37)
Some college	928 (31)
Bachelor’s degree or higher	782 (26)
Household income, $	
<24 999	621 (21)
25 000-74 999	1163 (39)
75 000-149 999	831 (28)
≥150 000	399 (13)
Marital status	
Married	1079 (36)
Widowed	149 (5)
Divorced	364 (12)
Separated	66 (2)
Never married	1357 (45)
Employment status	
Working full-time	1596 (53)
Working part-time	340 (11)
Not working	1079 (36)
Current or former military	380 (13)
Health insurance	2697 (90)
Metropolitan area residence	2756 (91)
Region of residence	
Northeast	513 (17)
Midwest	484 (16)
South	1700 (57)
West	317 (10)
Children living in home, mean (SD), No.	0.64 (1.07)

Table 2 and Table 3 show the results for individual GVEs. In the analytic sample of 2924 respondents, reporting being threatened with a firearm was significantly associated with reporting a lifetime history of suicidal ideation (odds ratio [OR], 1.44; 95% CI, 1.01-2.05; *P* = .04). Reporting ever knowing someone who has been shot was associated with an increased risk for reporting lifetime suicidal ideation (OR, 1.44; 95% CI, 1.05-1.97; *P* = .02). Additional results for suicidal ideation models split by passive vs active ideation are available in eAppendix 2 in Supplement 1. None of the individual exposure types was associated with past-year or past-month suicidal ideation. In the subsample of 617 respondents who experienced suicidal ideation, reporting being shot with a firearm was associated with a significant increase in reporting ever making preparations for a suicide attempt (OR, 3.73; 95% CI, 1.10-12.64; *P* = .03). Reporting being threatened with a firearm (OR, 2.41; 95% CI, 1.14-5.09; *P* = .02) and knowing a family member or friend who has been shot (OR, 2.86; 95% CI, 1.42-5.74; *P* = .003) were each associated with reporting ever attempting suicide.

**Table 2.** Individual Gun Violence Exposures and Suicidal Ideation Among 2924 Respondents^a^

Gun violence exposure	Lifetime suicidal ideation		Past-year suicidal ideation		Past-month suicidal ideation	
	OR (SE) [95% CI]	*P* value	OR (SE) [95% CI]	*P* value	OR (SE) [95% CI]	*P* value
Threatened with a gun	1.44 (0.26) [1.01-2.05]	.04	1.07 (0.26) [0.67-1.71]	.77	1.50 (0.51) [0.77-2.90]	.23
Shot with a gun	0.86 (0.28) [0.46-1.61]	.63	0.61 (0.35) [0.20-1.88]	.38	0.34 (0.26) [0.08-1.54]	.16
Family or friend shot	1.44 (0.23) [1.05-1.97]	.02	1.18 (0.24) [0.79-1.76]	.41	0.90 (0.29) [0.48-1.69]	.74
Witnessed or heard about a shooting	1.11 (0.17) [0.82-1.49]	.50	1.16 (0.22) [0.80-1.69]	.44	1.19 (0.32) [0.70-2.02]	.52

Abbreviation: OR, odds ratio.

^a^ All models controlled for self-rated health, sex, age, educational level, household income, marital status, employment status, military status, number of children living in the home, insurance status, metropolitan area residence, and US region.

**Table 3.** Individual Gun Violence Exposures and Suicidal Behaviors in a Subsample of 617 Respondents^a^

Gun violence exposure	Suicide attempt preparation		Attempted suicide	
	OR (SE) [95% CI]	*P* value	OR (SE) [95% CI]	*P* value
Threatened with a gun	1.55 (0.54) [0.79-3.06]	.20	2.41 (0.92) [1.14-5.09]	.02
Shot with a gun	3.73 (2.32) [1.10-12.64]	.03	0.71 (0.53) [0.17-3.05]	.64
Family or friend shot	1.24 (0.45) [0.61-2.53]	.54	2.86 (1.01) [1.42-5.74]	.003
Witnessed or heard about a shooting	0.84 (0.29) [0.43-1.65]	.61	0.66 (0.24) [0.32-1.35]	.25

Abbreviation: OR, odds ratio.

^a^ All models controlled for self-rated health, sex, age, educational level, household income, marital status, employment status, military status, number of children living in the home, insurance status, metropolitan area residence, and US region.

Table 4 and Table 5 show the results for cumulative GVE. Exposures to 1 (OR, 1.69; 95% CI, 1.19-2.39; *P* = .003) and 2 (OR, 1.69; 95% CI, 1.17-2.44; *P* = .005) types of gun violence were each associated with increased lifetime reporting of suicidal ideation. Being exposed to 3 or more types of gun violence was associated with the greatest odds of lifetime reporting for suicidal ideation (OR, 2.27; 95% CI, 1.48-3.48; *P* < .001). We found no significant associations for suicidal ideation in the past year or month. Being exposed to 3 or more types of firearm violence was associated with reporting ever making preparations for a suicide attempt (OR, 2.37; 95% CI, 1.01-5.63; *P* = .05). Exposure to 2 (OR, 4.78; 95% CI, 1.80-12.71; *P* = .002) and 3 (OR, 4.01; 95% CI, 1.41-11.44; *P* = .009) types of gun violence was associated with reporting ever attempting suicide.

**Table 4.** Cumulative Gun Violence Exposure and Suicidal Ideation Among 2924 Respondents^a^

Gun violence exposures	Lifetime suicidal ideation, OR (SE) [95% CI]	*P* value	Past-year suicidal ideation, OR (SE) [95% CI]	*P* value	Past-month suicidal ideation, OR (SE) [95% CI]	*P* value
1 Type	1.69 (0.30) [1.19-2.39]	.003	1.21 (0.28) [0.77-1.89]	.41	1.18 (0.41) [0.60-2.32]	.63
2 Types	1.69 (0.32) [1.17-2.44]	.005	1.05 (0.26) [0.64-1.72]	.84	0.88 (0.31) [0.44-1.76]	.71
≥3 Types	2.27 (0.49) [1.48-3.48]	<.001	1.53 (0.44) [0.87-2.68]	.13	1.48 (0.60) [0.67-3.26]	.32

Abbreviation: OR, odds ratio.

^a^ All models controlled for self-rated health, sex, age, educational level, household income, marital status, employment status, military status, number of children living in the home, insurance status, metropolitan area residence, and US region.

**Table 5.** Cumulative Gun Violence Exposure and Suicidal Behaviors in a Subsample of 617 Respondents^a^

Gun violence exposures	Suicide attempt preparation		Attempted suicide	
	OR (SE) [95% CI]	*P* value	OR (SE) [95% CI]	*P* value
1 Type	1.90 (0.75) [0.87-4.14]	.10	1.51 (0.77) [0.55-4.14]	.42
2 Types	2.15 (0.87) [0.96-4.78]	.06	4.78 (2.38) [1.80-12.71]	.002
≥3 Types	2.37 (1.04) [1.01-5.63)	.05	4.01 (2.14) [1.41-11.44]	.009

Abbreviation: OR, odds ratio.

^a^ All models controlled for self-rated health, sex, age, educational level, household income, marital status, employment status, military status, number of children living in the home, insurance status, metropolitan area residence, and US region.

## Discussion

Research demonstrates that the pernicious effects of GVE disproportionately burden Black communities.^8,26^ Black individuals in the US experience intersecting systems of structural discrimination that affect the risk of exposure to gun violence and related health outcomes as well as inhibit access to critical resources, such as high-quality health care and culturally congruent therapeutic services to address the harms associated with GVE. The primary aim of this study was to extend existing research by examining the association between GVE and suicidal thoughts and behaviors within a nationally representative sample of Black adults.

Past research suggests an association between GVE and risk for suicidal ideation, although these studies did not focus on Black individuals in the US, were limited to specific populations (eg, young adults experiencing homelessness),^27^ and used measures that captured limited GVEs.^16^ We found that specific forms of GVE (eg, being threatened, knowing a family member or friend who has been shot) and cumulative GVE were associated with lifetime suicidal thoughts among Black US adults. Notably, exposure to 3 or more forms of gun violence was associated with a substantially increased likelihood of reporting lifetime suicidal thoughts, suggesting that experiencing numerous forms of gun violence is associated with an individual’s propensity to consider suicide. Gun violence exposure was not associated with past-month or past-year suicidal thoughts, although this is likely because our data were not ideal for detecting precision in the timing of these associations.

Our data could not identify mechanisms to explain the path from cumulative GVE to suicidal thoughts. However, 1 possibility is that exposures to such traumatic experiences are associated with increased hopelessness and diminished connections (social network and broader community) and sense of value (eg, diminished employment and wealth generation opportunities). Leading theories of suicide, such as the Three Step Theory of Suicide,^30,31^ posit that when individuals feel less connected to others, when their experiences of pain exceed their sense of connection, and when they feel hopeless that the situation will change, they are at dramatically increased risk of experiencing suicidal thoughts. However, many modern theories of suicide have not been created specifically to examine suicide among racial and ethnic minoritized populations, underscoring the need to consider how systemic racism and discrimination faced by Black individuals in the US affect any potential mechanisms linking GVE and outcomes related to suicide.^32^ The pervasive social stigma associated with suicide and psychiatric illness within Black communities may further compound these factors and serve as a significant barrier to receiving psychiatric treatment.^33^

Importantly, the Three Step Theory of Suicide notes that capacity to transition from suicidal ideation to action can be influenced by repeated exposure to painful and provocative events.^31^ Within this context, our findings suggest that GVE is associated with specific suicidal behaviors, underscoring that GVE may act not only as a facilitator of suicidal thought but also as a factor associated with decisions to act on such thoughts. We are cautious in our interpretations of the behavior-specific findings given the relatively small subsample, however, and underscore the need for data that rely on longitudinal, community-informed designs to assess the specific transition from suicidal ideation to action associated with GVE.

Research has demonstrated that GVE is associated with exacerbated disparities in socioeconomic opportunity and collective well-being.^34^ Our results suggest that primary prevention of firearm violence may be a critical avenue for reducing suicidal ideation and behavior among Black US adults. Yet, it is also necessary to make culturally congruent resources available for those who have directly or vicariously been exposed to gun violence alongside opportunities for broader collective healing given the current absence of comprehensive prevention.^35^ Prevention and resource availability are particularly urgent for those living in racial and ethnic minoritized communities.

Although we could not assess for racial disparities in GVE and suicide-related outcomes, there is a need for continued research in this area. Prior work suggests, for instance, that Black boys are at greatest risk for both GVE and depression^25^ and, among individuals who identify as Latinx or other race, there is a significant association between GVE and depression.^16^ Researchers should also work to examine the capacity of protective factors, such as access to particular health care resources or social support structures, to reduce the risk of suicidal ideation and behaviors associated with GVE among Black individuals and other racial and ethnic groups in the US. It is also necessary to assess how GVE conceptualized as a traumatic experience corresponds with greater risk for suicidal ideation and behaviors compared with other forms of trauma, such as experiencing a severe automotive crash or other types of assault (eg, sexual).^36,37^ A clearer understanding of these distinctions is critical to properly inform policy and clinical prescriptions for addressing gun violence as a distinct form of traumatic exposure.

### Limitations

Several limitations are worth noting. First, our findings were cross-sectional, and we could not directly assess the directionality of the effects or determine causality. The time anchors used for our main variables precluded full knowledge of how recency of GVE is associated with suicidal ideation and behavior. Second, the constructs of interest are relatively low-base-rate phenomena, and we were limited in our ability to assess and test relevant models. We were particularly cautious in interpreting effect sizes for suicidal behaviors in the subsample of those who exhibited suicidal ideation. Third, we could only examine associations among Black US adults, and future studies should aim to analyze disparities across racial and ethnic groups. Finally, our data only captured respondent sex, and models did not include gender identity or sexual orientation. Future studies should examine these factors given that sexual and gender minority groups are at elevated risk for suicide-related outcomes and experience higher rates of violence exposure.^38,39^

## Conclusions

In this cross-sectional study of a nationally representative sample of Black US adults, we centered the experiences of those living in communities disproportionately impacted by gun violence yet often underrepresented in academic research. By considering the association of GVE with suicide risk through the lens of the ideation-to-action framework, we were able to assess whether the cumulative experience of gun violence is associated with not only suicidal thoughts but also suicidal behavior. Overall, our findings suggest that GVE is significantly associated with suicidal thoughts and behavior. The disproportionate burden of GVE borne by Black communities and exacerbated by numerous structural inequities may represent an even more substantial injustice than previously understood, as it may be influencing suicide rates within those same communities.

## Notes

Supplement 1. **eAppendix 1.** Positionality Statement **eAppendix 2.** Passive vs Active Lifetime Suicidal Ideation Models

Supplement 2.
Data Sharing Statement
