# Supplementary material for: Gun Violence Exposure and Suicide Among Black Adults
Source: JAMA Netw Open. 2024 Feb 6;7(2):e2354953. doi: 10.1001/jamanetworkopen.2023.54953 (PMC10848043; doi:10.1001/jamanetworkopen.2023.54953)
Supplement: Supplement 1. — eAppendix 1. Positionality Statement eAppendix 2. Passive vs Active Lifetime Suicidal Ideation Models [file jamanetwopen-e2354953-s001.pdf]

Supplementary Online Content

Semenza DC, Daruwala S, Brooks Stephens JR, Anestis M. Gun violence exposure and suicide among Black adults. *JAMA Netw Open*. 2024;7(2):e2354953. doi:10.1001/jamanetworkopen.2023.54953

eAppendix 1. Positionality Statement

eAppendix 2. Passive vs Active Lifetime Suicidal Ideation Models

© 2024 Semenza DC et al. *JAMA Network Open*.

eAppendix 1. Positionality Statement

Of the four authors on this manuscript, two identify as white men and two identify as women of color. One of the white men conducts research on community health disparities related to interpersonal gun violence while the other conducts research on firearm suicide prevention. Of the two women of color, one identifies as a Black woman and her work has consistently focused on suicide risk and mental health within Black communities. The second woman of color identifies as a South Asian American woman and her works focuses on firearm suicide prevention.

Each of the authors recognize that their positionality undoubtedly impacted the manner in which they developed the study and interpreted the findings in different ways, even as they have continuously worked to develop inclusive environments in their workplaces and to ensure their work addresses the experiences and perspectives of communities that differ from their own. The entire authorship team worked together in the crafting of this document and are hopeful that their diverse perspectives resulted in an accurate and actionable manuscript.

© 2024 Semenza DC et al. *JAMA Network Open*.

eAppendix 2. Passive vs. Active Lifetime Suicidal Ideation Models

Table B1. Individual Gun Violence Exposures and Lifetime Suicidal Ideation (Passive vs. Active; N = 2,924)

	Passive		Active	
	OR (SE) [95% CI]	P value	OR (SE) [95% CI]	P value
Threatened w/ gun	1.34 (0.25) [0.94-1.94]	0.10	1.70 (0.43) [1.03-2.78]	0.03
Shot w/ gun	0.97 (0.31) [0.52-1.83]	0.92	0.58 (0.28) [0.22-1.49]	0.25
Family/friend shot	1.41 (0.23) [1.03-1.95]	0.03	1.91 (0.47) [1.19-3.09]	0.008
Witnessed/heard	1.15 (0.17) [0.85-1.54]	0.36	0.60 (0.07) [0.48-0.75]	0.65

Table B2. Cumulative Gun Violence Exposure and Lifetime Suicidal Ideation (Passive vs. Active; N = 2,924)

	Passive		Active	
	OR (SE) [95% CI]	P value	OR (SE) [95% CI]	P value
One type	1.66 (0.30) [1.16-2.36]	0.005	2.16 (0.57) [1.28-3.63]	0.004
Two types	1.59 (0.30) [1.10-2.31]	0.01	1.96 (0.54) [1.14-3.38]	0.01
Three or more types	2.28 (0.50) [1.48-3.52]	<0.001	3.13 (0.90) [1.78-5.52]	<0.001

Notes: All models control for self-rated health, sex, age, education, household income, marital status, employment status, military status, number of children living in the home, insurance status, metro area residence, and US region. Passive ideation includes the following items: “I wish I could disappear or not exist” “I wish I was never born” “My life is not worth living” “I wish I could go to sleep and never wake up” and “I wish I were dead.” Active ideation includes the following items: “Maybe I should kill myself” “I should kill myself” and “I’m going to kill myself.”

© 2024 Semenza DC et al. *JAMA Network Open*.
