# Supplementary material for: Gun Violence Exposure and Suicide Among Black Adults
Source: JAMA Netw Open. 2024 Feb 6;7(2):e2354953. doi: 10.1001/jamanetworkopen.2023.54953 (PMC10848043; doi:10.1001/jamanetworkopen.2023.54953)
Supplement: Supplement 2. — Data Sharing Statement [file jamanetwopen-e2354953-s002.pdf]

Data Sharing Statement

Semenza. Gun Violence Exposure and Suicide Among Black Adults. *JAMA Netw Open*. Published February 06, 2024. doi:10.1001/jamanetworkopen.2023.54953

Data
Data available: No

Additional Information
Explanation for why data not available: Data are currently under restricted access outside of the New Jersey Gun Violence Research Center at Rutgers University.
